# Cerebral Venous Sinus Thrombosis as an Unexpected Complication of COVID-19 Pneumonia

**DOI:** 10.7759/cureus.16498

**Published:** 2021-07-19

**Authors:** Mohammed Abdulgayoom, Elabbass Abdelmahmuod, Ahmed Elfaki, Malik A Halabiya

**Affiliations:** 1 Internal Medicine, Hamad Medical Corporation, Doha, QAT; 2 General Surgery, Hamad Medical Corporation, Doha, QAT

**Keywords:** cerebral venous sinus thrombosis, sars-cov-2, covid 19, sagittal sinus thrombosis, therapeutic anticoagulation

## Abstract

Coronavirus disease 2019 (COVID-19) is commonly associated with acute respiratory distress syndrome and acute cardiac and renal injuries. However, thromboembolic events are also prevalent in COVID-19. The pathogenesis of COVID-19 hypercoagulability is not well known but may be linked to the cytokine storm induced by a viral infection or endothelial damage that triggers a cascade leading to hypercoagulability. Because vascular endothelium has angiotensin-converting enzyme 2-like lung tissue, COVID-19 targets lung tissue and vascular endothelium, leading to thrombosis. We present a rare case of a young patient with COVID-19 who presented with thrombosis of the cerebral venous system managed with anticoagulation. This case highlights the need for heightened awareness of this atypical but potentially treatable complication of the COVID-19 disease spectrum.

## Introduction

The severe acute respiratory syndrome coronavirus 2 (SARS‑CoV‑2), the virus that causes coronavirus disease 2019 (COVID-19), has recently been linked to many hematologic issues, including an exceptionally high rate of venous thromboembolism (VTE). The pathogenesis of COVID-19 hypercoagulability is not fully understood, but it is hypothesized to be due to cytokine storm induced by a viral infection, and/or endothelial damage caused by the virus itself which can trigger a cascade that leads to hypercoagulability [1].

Cerebral venous sinus thrombosis (CVST) is a rare cause of stroke that primarily affects young people. The condition is uncommon, with an incidence of 0.32 to 1.57 per 100,000 person-years and a female-to-male ratio of 3:1 [2]. There are several risk factors for CVST, including congenital thrombophilia, oral contraceptives, autoimmune diseases such as systemic lupus erythematosus, antiphospholipid syndrome, infections, and malignancy. Infectious causes account for a small percentage of CVST cases in developed countries, but they are a significant cause in developing countries, accounting for 8.1%-35.6% of CVST cases [3].

While pulmonary embolism (PE) and deep venous thrombosis (DVT) have been well documented in patients with COVID-19, there is limited information on CVST associated with COVID-19 [4]. We present a case of CVST due to COVID-19 in a 30-year-old Indian man who presented to the ED with a headache one week after he was diagnosed with COVID-19 pneumonia.

## Case presentation

**Figure 1:**
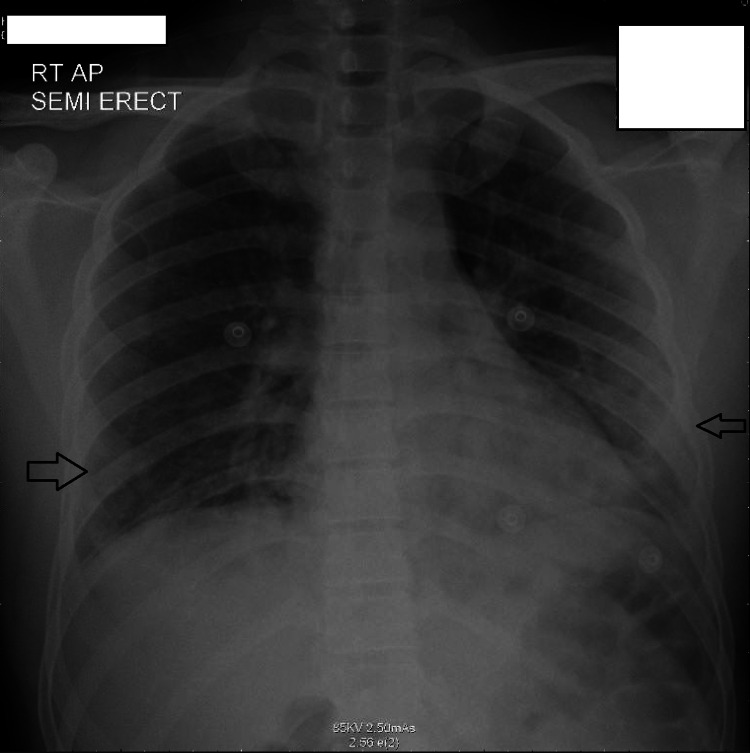
Chest X-ray showing multifocal airspace opacities noted in bilateral lower zone pattern of COVID-19 pneumonitis. COVID-19: Coronavirus disease 2019.

A 30-year-old Indian man, previously healthy, first presented to our hospital with fever, cough, and dyspnea diagnosed as COVID-19 pneumonia, depending on a SARS-CoV-2-positive nasal polymerase chain reaction (PCR) and chest X-ray findings (Figure 1).

He stayed in the hospital for three days on oxygen. His maximum oxygen requirement was 2L via nasal cannula to maintain an oxygen saturation of >93%. He was also treated with enoxaparin (40 mg for DVT prophylaxis), dexamethasone (6 mg orally), hydroxychloroquine, and lopinavir/ritonavir.

On the third day after admission, the patient was doing fine, was afebrile, and maintained adequate oxygen saturation on room air. Therefore, he was discharged to a quarantine facility. Four days later, the patient presented again to the hospital reporting concerns of a headache and vomiting lasting three days and an episode of brief loss of consciousness on the day of presentation. He reported that his headache was severe, with a score of 8/10 on the pain scale, and described as generalized, throbbing, and continuous with no aggravating factor. His headache was associated with vomiting more in the morning and photophobia, and it was slightly relieved by paracetamol.

On the day of presentation, he developed a brief loss of consciousness for approximately three minutes associated with a jerky movement of all limbs and urine incontinence and followed by sleepiness for less than an hour. There was no fever, no limb weakness, and no other symptoms.

On physical examination, we noted that the patient looked sick; in pain; avoiding lights; sleepy but conscious; oriented to time, place, and persons; and had negative meningeal signs. We saw no focal neurological deficit, and the findings from the remainder of the examination were unremarkable.

His laboratory test results are presented in Table 1 and were significant for leucocytosis (mainly neutrophilia). His D-dimer test was negative on the first admission and was not repeated on the second admission. His renal function and electrolyte levels were within reference ranges. The liver function test results were also within reference ranges apart from slightly increased alanine aminotransferase and aspartate transaminase. We did not conduct a thrombophilia workup.

**Table 1:** Laboratory test results. ANC: Absolute neutrophil count; ALP: Alkaline phosphatase; ALT: Alanine aminotransferase; AST: Aspartate aminotransferase; CRP: C-reactive protein; INR: International normalized ratio.

Analyte	Result	Reference range
Hemoglobin	15.3 g/dl	13-17 g/dl
WBCs	14.4 x 10^3/µL	4-10 x 10^3/µL
Platelets	368 x 10^3/µL	150-450 x 10^3/µL
ANC	11.9 x 10^3/µL	2-7 x 10^3/µL
Lymphocyte	1.1 x 10^3/µL	1-3 x 10^3/µL
Urea	7.5 mmol/L	2.5-7.8 mmol/L
Creatinine	57 µmol/L	64-110 µmol/L
Sodium	133 mmol/L	135-145 mmol/L
Potassium	4.8 mmol/L	3.5-5.2 mmol/L
Albumin	34 gm/L	35-45 g/L
ALP	61 U/L	40-150 U/L
ALT	358 U/L	0-55 U/L
AST	106 U/L	4-34 U/L
Bilirubin	12 µmol/L	3.4-20.5 µmol/L
CRP	7 mg/L	<5 mg/L
Procalcitonin	0.1 ng/mL	<1 ng/mL
INR (on admission)	1.2	1
INR (on discharge)	2.4	1

**Figure 2:**
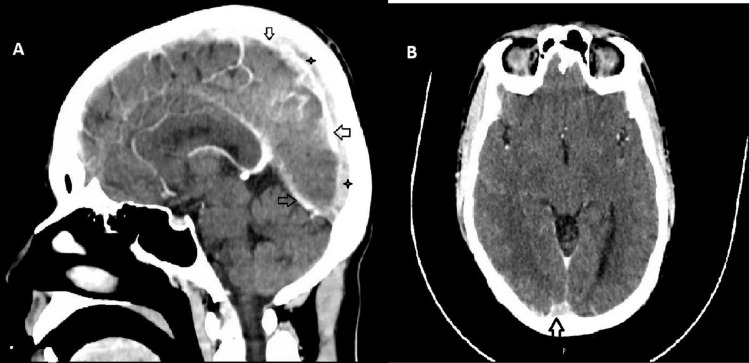
CT scan. (A) Sagittal view. (B) Axial view. Superior sagittal sinus and its tributaries show high density (arrow) with filling defect (star) on post-contrast examination suggesting dural sinus thrombosis.

A CT scan of his head (Figure 2) showed superior sagittal sinus and its tributaries, right transverse, sigmoid sinuses, and right internal jugular vein with a high-density filling defect seen on post-contrast examination, suggestive of dural sinus thrombosis. An MRI and venography confirmed the CT scan findings.

The patient was started on enoxaparin and levetiracetam. Within three days, he reported total resolution of the symptoms. Before discharge, warfarin was started, and the patient was discharged on both warfarin (to maintain his international normalized ratio between two to three for at least three months with regular follow-up) and levetiracetam with neurology follow-up.

## Discussion

COVID-19 is characterized by an exaggerated inflammatory response that, in some patients, can lead to extreme manifestations such as adult respiratory syndrome, sepsis, coagulopathy, and death [5]. A sagittal sinus thrombosis is a form of cerebral sinus thrombosis that is relatively uncommon. It happens when a blood clot blocks the brain's venous drainage system, causing a rise in intracranial pressure. The most common cause of cerebral sinus thrombosis is thrombophilia, and it can manifest in a wide range of neurologic signs and symptoms [6].

Coagulopathy is linked to a variety of viral infections. Viruses that activate endothelial cells directly or indirectly can impair coagulation and fibrinolytic systems. Antiphospholipid antibodies have also been found in immunocompetent patients with many viral infections, including cytomegalovirus, HIV, and varicella-zoster virus. Patients with human herpesviruses (DNA viruses) and HIV are believed to be hypercoagulable. A 10-fold increased risk of venous thrombosis has been linked to chronic HIV infection [6].

Patients with extreme COVID-19 symptoms are at high risk to develop thrombosis. The pathogenesis of thrombosis related to COVID-19 is not well understood. However, there are several hypotheses including an increase in the vasoconstrictor angiotensin-II, a decrease in the vasodilator peptide angiotensin-(1-7), and the sepsis-induced release of cytokines [7]. Other factors include direct viral effects on endothelial cells, resulting in excessive thrombin production and fibrinolysis inhibition. Hypoxia is also linked to increased blood viscosity and the activation of hypoxia-related genes that regulate coagulation and fibrinolysis, making thrombotic events more likely [6].

In severe COVID-19 patients, the significant increase in D-dimer is the most critical change in coagulation parameters, and increasingly higher values can be used as a prognostic parameter for a worse outcome [8]. Therapeutic anticoagulation is the standard treatment of thrombosis and PE. Unfractionated heparin is commonly preferred for treating VTE in ICUs. However, frequent monitoring is required [9]. Longer-acting agents like low-molecular-weight heparin may also be considered. It can be given subcutaneously once or twice per day and does not need to be monitored often to ensure it is dosed correctly [7].

Oral anticoagulants, such as warfarin, the direct thrombin inhibitor dabigatran, and factor Xa inhibitors like apixaban and rivaroxaban, are good options for anticoagulation after discharge [10]. VTE and thrombosis prophylaxis are vital to therapy for COVID-19, and these options should be considered for high-risk patients even after discharge from the hospital [6].

Few cases report the association between CVST and COVID-19. While the incidence of COVID-19 is more commonly associated with acute respiratory distress syndrome and acute cardiac and renal injuries, thromboembolic incidents are becoming more common [7].

## Conclusions

COVID-19 is a significant contributor to hypercoagulation and rising disease fatality. We discussed the case of a young patient with COVID-19 who developed thrombosis of the cerebral venous system managed with anticoagulation therapy. This case highlights the need for acute awareness among physicians of this atypical but potentially treatable complication of COVID-19.
